# Possible Predictors of Involuntary Weight Loss in Patients with Alzheimer’s Disease

**DOI:** 10.1371/journal.pone.0157384

**Published:** 2016-06-27

**Authors:** Massimo Venturelli, Emiliano Cè, Eloisa Limonta, Ettore Muti, Renato Scarsini, Anna Brasioli, Federico Schena, Fabio Esposito

**Affiliations:** 1 Department of Biomedical Sciences for Health, University of Milan, via Colombo 71, 20133, Milan, Italy; 2 Mons. Mazzali Foundation, via Trento 10, 46100, Mantua, Italy; 3 Department of Neurological and Movement Sciences, University of Verona, via Casorati 43, 47131, Verona, Italy; Nathan Kline Institute and New York University School of Medicine, UNITED STATES

## Abstract

Loss in body mass (∆BM) is a common feature in patients with Alzheimer’s disease (AD). However, the etiology of this phenomenon is unclear. The aim of this cohort study was to observe possible ∆BM in AD patients following a standard institutionalized diet. Secondary objective was to identify possible predictors of ∆BM. To this end, 85 AD patients (age: 76±4 yrs; stature: 165±3 cm; BM: 61.6±7.4 kg; mean±standard deviation) and 86 controls (CTRL; age: 78±5 yrs; stature: 166±4 cm; BM: 61.7±6.4 kg) were followed during one year of standard institutionalized diet (~1800 kcal/24h). BM, daily energy expenditure, albuminemia, number of medications taken, and cortisolism, were recorded PRE and POST the observation period. Potential predictors of ∆BM in women (W) and men (M) with AD were calculated with a forward stepwise regression model. After one year of standard institutionalized diet, BM decreased significantly in AD (-2.5 kg; p < 0.01), while in CTRL remained unchanged (-0.4 kg; p = 0.8). AD patients and CTRL exhibited similar levels of daily energy expenditure (~1625 kcal/24h). The combination of three factors, number of medications taken, albuminemia, and cortisolism, predicted ∆BM in W with AD. At contrary, the best predictor of ∆BM in M with AD was the cortisolism. Despite a controlled energy intake and similar energy expenditure, both W and M with AD suffered of ∆BM. Therefore, controlled diet did not prevent this phenomenon. The assessments of these variables may predict W and M with AD at risk of weight loss.

## Introduction

Changes in body composition, such as loss in total body mass (BM) affect adversely physical function and quality of life of patients with Alzheimer’s disease (AD) [1]. Such a change could be the result of an unbalance between energy intake and energy expenditure [2]. For instance, weight loss (WL) can be prevented in patients with dementia by increasing their energy intake [3]. On the other side, a murine study suggested that AD is associated with a hyper-metabolic state, which may affect daily energy expenditure (DEE) and body composition [4]. Conversely, the resting metabolic rate (RMR) in humans was found to be similar in patients with AD and in healthy old individuals [5]. Moreover, patients with AD usually demonstrate an increased level of daily physical activity (PA) induced by behavioral disorders, such as wandering, that may likely increase the DEE [6].

Given their chronic treatment with medications, the potential contribution of pharmacological therapy in patients with AD to the aforementioned changes in body composition has been recently debated [2, 7]. Recognized side effects of medications, such as antidepressants, cholinesterase inhibitors, and corticosteroids drugs, include gastrointestinal symptoms, such as nausea, vomiting and diarrhea [8] that may affect as well BM. Moreover, pharmacological interactions may potentially increase the side effects affecting body composition.

Strong evidence indicates that AD is associated with a dysregulation of the hypothalamic–pituitary–adrenal axis (HPA-axis) [9], with a clear increase in cortisol levels [10]. The effects of hypercortisolism are wide-ranging, affecting widely the skeletal muscle, thus leading to a remarkable sarcopenia.

Lastly, growing literature supports the theory that oxidative damage is associated with the pathogenesis and development of AD [11]. It was recognized that albumin plasma levels, the main antioxidants plasma carrier, are significantly reduced in patients with AD [12]. Additionally, elevated oxidative stress markers are normally associated with the increased numbers of neutrophils in patients with AD [13]. However, their role in the WL remains not clear.

Therefore, the aim of the present cohort study was to observe possible BM changes in AD patients following a standard institutionalized diet. Secondary aim was to determine the contribution of energy intake and expenditure, reduction in antioxidants plasma carrier, pharmacological treatments, hypercortisolism, and chronic inflammation to the WL in patients with AD. The assessment of these values may help to predict this challenging clinical phenomenon.

## Methods

### Study design

**Fig 1:**
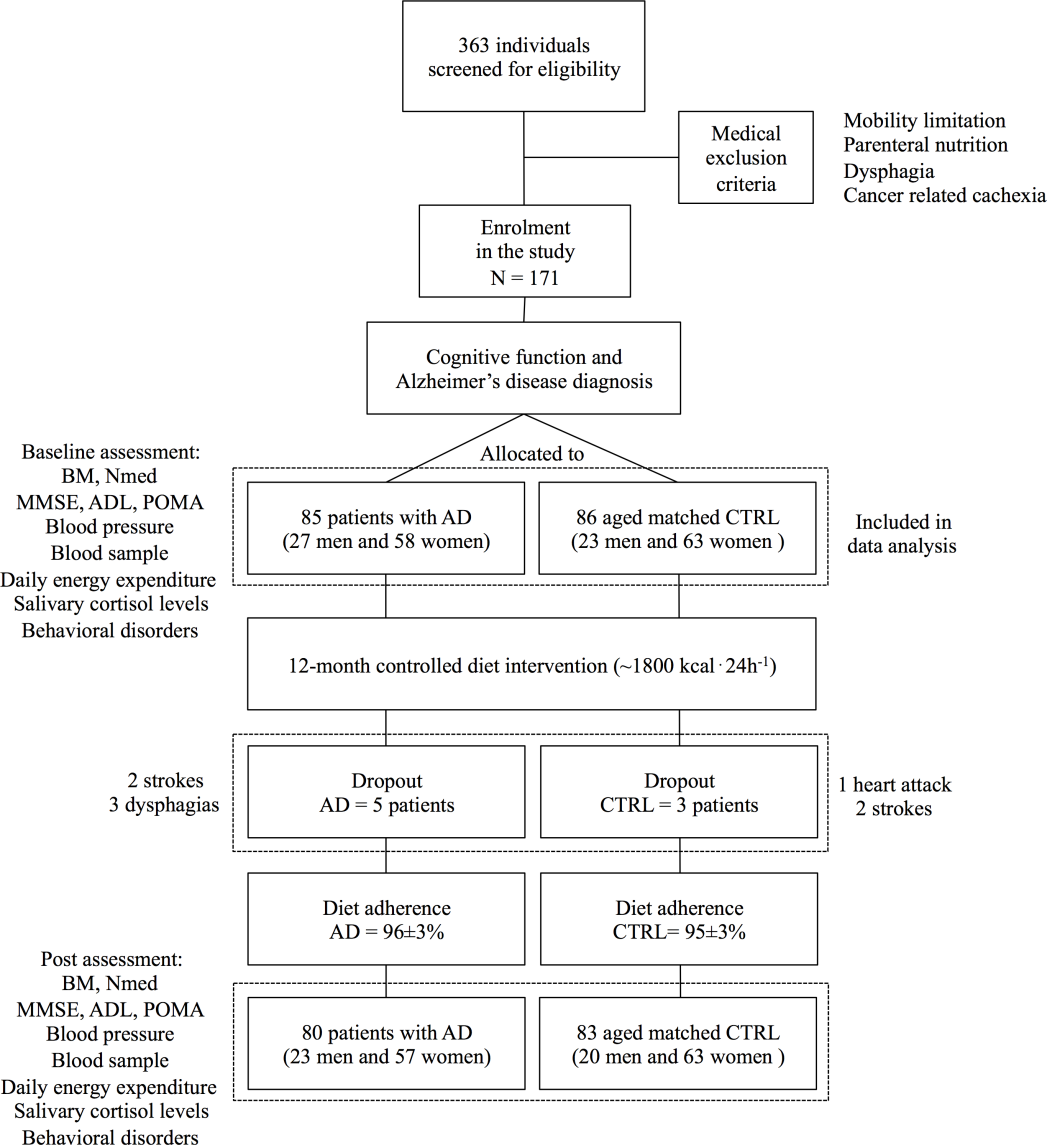
Participant flow diagram. AD, Alzheimer’s disease; CTRL, controls; BM, body mass; Nmed, number of medications; MMSE, Mini-Mental State Examination; ADL, activities of daily life; POMA; performance-oriented assessment of mobility.

This cohort study compared the effects of 12 months standard institutionalized diet in a group of patients with AD and age-matched controls (CTRL) without symptoms of dementia (Fig 1 and S1 File). BM, mobility performance, cognitive function, blood pressure, blood sample, daily energy expenditure, and salivary cortisol levels were assessed at baseline and after 12-month of standard institutionalized diet.

### Participants

Eligible volunteers were selected among nursing home residents of Alzheimer’s care units and standard geriatrics care units at the Mons. Mazzali Geriatric Institute in Mantua, Italy. Inclusion criteria were an age >65 yrs. Being both energy expenditure and intake two key factors analyzed in this study, patients with mobility limitations (performance-oriented assessment of mobility problems, POMA) [14] score lower than 25, and subjects with nutritional support (parenteral nutrition), were not included in the study. Moreover, patients with dysphagia, cancer related cachexia, were not selected for the study.

### Screening

From October 2013, three hundred sixty-three volunteers were screened for eligibility, 171 subjects met the above mentioned inclusion criteria. Eighty-five residents of the Alzheimer’s care units (27 men and 58 women) with a confirmed diagnosis of Alzheimer’s diseases dementia, and 86 residents of standard geriatrics care units (CTRL, 23 men and 63 women) without clinical evidence of dementia, were selected for the study. Selected participants were than monitored from January 2014 to January 2015. Patients’ relatives or legal representatives and CTRL received written and oral information about the study before signing the informed consent form. The University of Milan ethical committee approved the study (approval number 63/14). The study was carried out in accordance with the Declaration of Helsinki (2013) of the World Medical Association for experiments involving humans. Characteristics of the participants are reported in Table 1.

**Table 1:** Demographic and clinical characteristics of the study participants at baseline. Values are expressed as mean ± standard deviation (or percentage in brackets).

Characteristic	CTRL	AD
	(N = 86)	(N = 85)
**Age—yrs**	78 ± 5	76 ± 4
**Female sex—no. (%)**	63 (73)	58 (68)
**Body mass index (kg∙m**^**-2**^**)**	23 ± 3	23 ± 4
**Duration of disease—yrs**	-	8 ± 2
**Duration of nursing home living—yrs**	3.9 ± 2.1	3.2 ± 2.5
**Participants taken medications–no. (%)**		
**Quetiapine**	1 (1)	29 (32) *
**Citalopram**	8 (9)	26 (30) *
**Donepezil**	-	6 (7) *
**Memantine**	-	2 (2) *
**Ticlopidin**	3 (3)	12 (14) *
**Furosemide**	4 (4)	15 (18) *
**Bisoprolol**	15 (17)	12 (14)
**Rabeprazol**	24 (28)	35 (41)
**Acetylsalicylic acid**	44 (51)	26 (30) *
**Mean number of medications–no.**	3 ± 1	6 ± 1 *
**Severity of dementia (CDR 0–3)**	-	2 ± 1
**Behavioral disturbance (NPI)**	-	68 ± 19
**Coexisting chronic conditions—no. (%)**		
**0**	4 (5)	5 (5)
**1**	14 (16)	19 (23)
**≥2**	68 (79)	61 (72)

* P<0.05 *vs* CTRL.

### Test procedures

Primary and secondary outcome measures were assessed at baseline and after 12-month of standard institutionalized diet. The medical staff monitored the category and total number of medications (N_MED_) taken by the participants.

### Controlled diet

During the 12-month standard institutionalized diet, 5 balanced meals per day (~1800 kcal⋅24h^-1^) were offered to the participants. It is important to note that the diet adopted for this observational study was part of their usual care, and was defined by a professional nutritionist of the Mons. Mazzali Foundation following the national guidelines of the Italian Department of Health for institutionalized patients [15]. More in details, breakfast (served at 7.00 AM) consisted of 200 ml of skimmed milk, 50 g of bread with two teaspoons of jam or honey; morning snack (served at 10.00 AM) contained 125 g of yogurt and cereals, or one fresh fruit (free choice among banana, apple or peach); lunch (served at noon) was composed by 80 g of pasta or rice, 100 g of meat or fish and one cup of fresh vegetables with lite dressing, such as olive oil; afternoon snack (served at 4.00 PM) consisted in a cup of tea with three or four dry cookies, or one fresh fruit (free choice among banana, apple or peach); dinner (served at 7.00 PM) comprised 100 g of soup, 50 g of bread, 100 g of meat or fish and half a cup of boiled vegetables with lite dressing.

### Adherence to the standard institutionalized diet

Nurses, caregivers and relatives assisting the patient’s during breakfast, lunch, and dinner were instructed to record into a database file any discrepancy between offered and eaten meals. To calculate the adherence to the diet, the amount of calories for additional or not eaten meals was estimated by weighting the food. Percent difference between offered and eaten meals was than calculated for each participant. Values of daily energy intake reported in the text represent the amount of calories calculated from the daily eaten meals.

### Primary outcome

Fasted BM was measured in the morning with a professional mechanical scale fitted with a stadiometer (Seca mod. 713; III-M; Seca Medical Scales and Measuring Systems, Birmingham, UK).

### Secondary outcomes

Clinical Dementia Rating (CDR) [16], Mini-Mental State Examination (MMSE) [17], and Neuro-Psychiatric Inventory (NPI) [18] were utilized to determinate progression and severity of dementia, as well as behavioral disorders. Nursing home residents’ gait and balance performance were assessed using the POMA [14]. Independence and level of activities of daily life (ADL) were evaluated with the Barthel index [19]. Fasted venous blood samples were analyzed for glucose, red blood cells, hemoglobin, high- and low-density lipoprotein blood levels, creatinine, azotemia, and hepatic function by standard techniques. Inflammation state was estimated from white blood cells count (WBC) and the percent difference in neutrophils, eosinophils, basophils, and lymphocytes. Plasma level of albumin (ALB) was utilized for the determination of antioxidants carrier. Blood level of retinol was utilized to control the effectiveness of the diet and the nutritional state of the participants. Levels of cortisol (COR) were measured via saliva samples using plain Sarstedt Salivette collection devices (Nümbrecht, Germany). Samples were collected at 6.30 AM, 11.30 AM, and 6.30 PM. Immediately after collecting the saliva samples, the Salivette tubes were centrifuged for 2 min at 1,000 rpm. Purified saliva were stored in a freezer at -20°C, and subsequently analyzed. COR levels were determined by a time-resolved immunoassay with fluorometric detection. For comparisons, each time point of COR levels were utilized, as well as the daily average level. DEE was measured with an Actiheart device (CamNtech, Cambridge, UK) allowing heart rate and acceleration data to be simultaneously recorded for 24 h/day for 7 consecutive days. The PA and the RMR components of daily energy expenditure were then calculated with a specific algorithm [20].

### Predictors of BM changes in patients with AD

To evaluate potential demographic covariant of pre-to-post changes in BM (∆BM), secondary outcomes assessed at baseline in patients with AD has been described with sex- and age-adjustment (less than 75 yrs; between 75 and 80 yrs; more than 80 yrs). Subsequently, being only the sex a potential covariant for ∆BM, in W and M with AD a univariate Pearson’s correlation model was performed to evaluate the single correlation between each baseline parameters. Secondary outcomes assessed at baseline significantly correlated with ∆BM has been considered potential predictors of involuntary weight loss for W and M with AD. The interaction between potential predictors of ∆BM in W and M with AD was then calculated with a forward stepwise regression model.

### Statistical analysis

Raw data were analyzed using a statistical software package (StatPlus, AnalystSoft Inc.—statistical analysis program for Mac OS®. Version v6. See http://www.analystsoft.com/en/). To check the normal distribution of the sampling, a Shapiro-Wilk test was applied. Whether this test failed, a non-parametric approach was applied. In consideration of preliminary results on salivary cortisol, hematological and blood chemistry measurements, and energy expenditure calculation, a sample size of 80 participants was selected to ensure a statistical power higher than 0.80 and a type 1 error of <0.05. Intention to treat approach was utilized to analyze the experimental data: missing values (participants dropout) were considered in the statistical analysis. A two-way (group x time) analysis of variance (ANOVA) was used to establish statistical differences among conditions. When necessary, a Bonferroni post-hoc, with Bonferroni correction test, was used to determine specific differences and interactions among variables A Kruskal Wallis ANOVA on ranks was applied to establish statistical differences among conditions presenting a non-normal distribution. Significance level was set at α<0.05. Results are presented as mean ± standard deviation (SD).

## Results

### Characteristics of the participants

363 individuals were screened for eligibility; 171 qualified (85 residents of the Alzheimer’s care units with a confirmed diagnosis of Alzheimer’s diseases and 86 without clinical evidence of dementia) were followed during the standard institutionalized diet. Table 1 displays the baseline characteristics of the study population. At baseline groups were matched for age, sex, body mass index (BMI), and coexisting chronic conditions. According to the CDR scale patients with AD were at the 2^nd^ stage of the dementia development, with an average time from Alzheimer’s disease diagnosis of 8 ± 2 years. Antidementia drugs and other medications taken by the two groups are displayed in Table 1.

### Adherence to the standard institutionalized diet and health-related dropout

**Fig 2:**
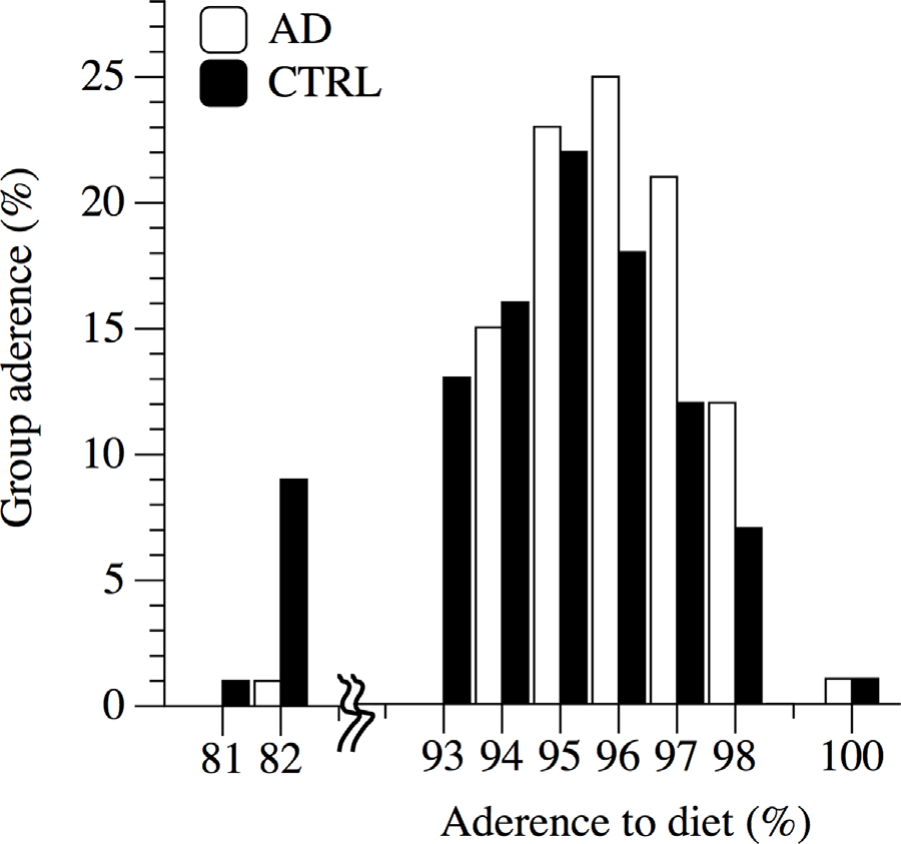
Distribution of diet-adherence in patients with AD and CTRL.

No adverse events related to the standard institutionalized diet were observed. Distribution of diet-adherence in patients with AD and CTRL is represented in Fig 2. Mean adherence was 96 ± 3% in the group of AD, and 95 ± 3% in the CTRL group, respectively (p = 0.9). During the observation period, no changes in the category of medications taken by the AD and CTRL were recorded. The follow-up time of the present cohort study was 372 ± 12 days, from January 2014 to January 2015. Five patients with AD (6% of the AD group) left the study to serious medical condition (2 strokes and 3 dysphagias). Three participants of the control group (4% of the CTRL group) also left the program because of serious health problems (1 heart attack and 2 strokes). In line with the intention to treat statistical approach, PRE values of dropouts were included for comparisons.

### Primary outcome

**Fig 3:**
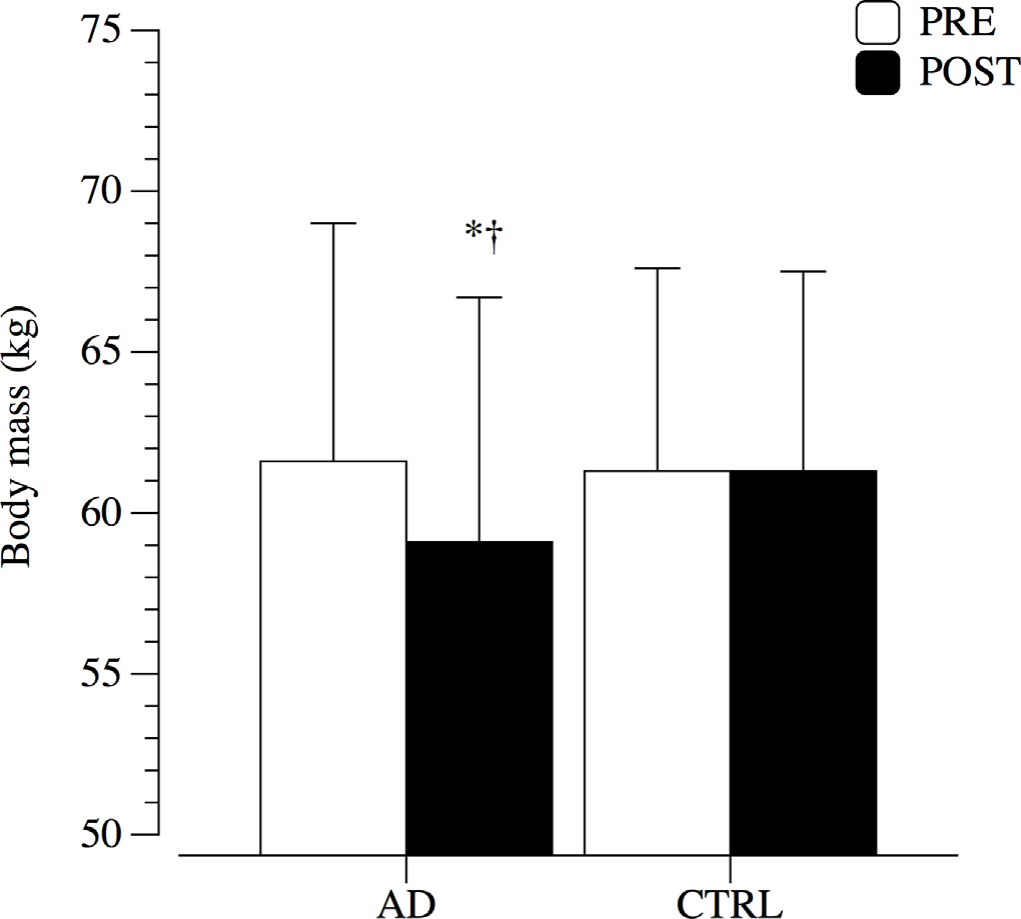
body mass at baseline (PRE) and after 12 months (POST) of controlled diet. *P<0.05 vs PRE; †P<0.05 vs CTRL.

Mean (± SD) inter- and intra-group differences in primary outcomes at baseline and 12 months are shown in Fig 3. After intervention, patients with AD demonstrated a significantly reduction in BM (-2.5 kg; p < 0.01), while in CTRL remained unchanged (-0.4 kg; p = 0.8).

### Secondary outcomes

**Fig 4:**
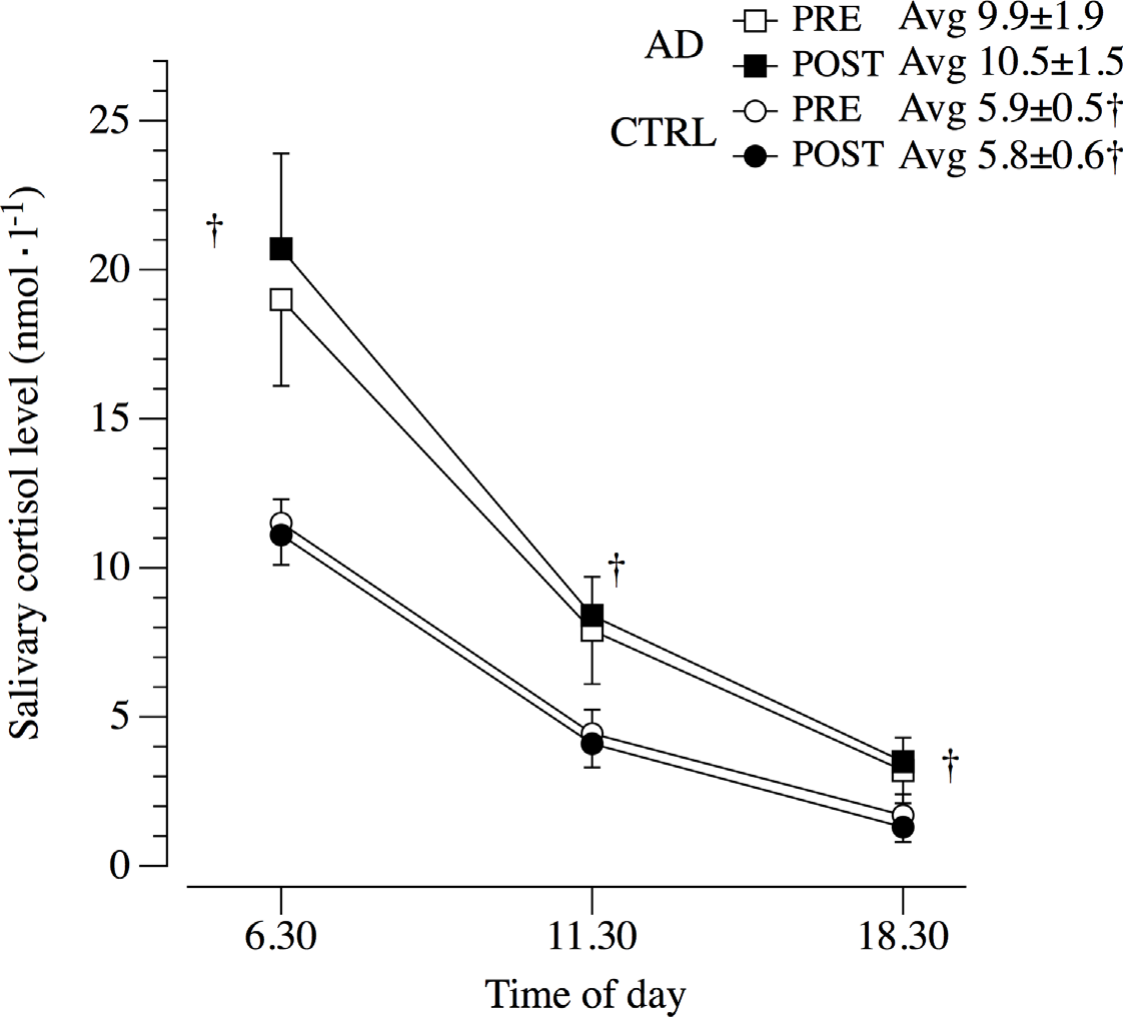
Circadian secretion of salivary cortisol. AD, Patients with Alzheimer’s disease; CTRL, aged matched controls; Avg, daily averaged cortisol. †P<0.05 vs CTRL.

After the standard institutionalized diet none of the selected secondary outcomes and hematological and blood chemistry measurements were significantly changed in the group of AD patients and CTRL (Table 2). However, AD patients demonstrated at baseline and after the period of the standard institutionalized diet, a chronic inflammatory state revealed by the elevated levels of WBC (p < 0.01), primarily due to the increased fraction of Neutrophil (Table 3). ALB plasma levels in AD were lower than in CTRL both at baseline (p < 0.01) and after the observation period (p < 0.01). Retinol blood level was similar between the two groups (Table 3). COR levels were significantly higher in the AD group at each time point (6.30, 11.30 and 18.30) compared to CTRL at both baseline and after the observation period (Fig 4). Systolic blood pressure was 5.2 mmHg higher in the AD patients at baseline; this difference was maintained (+4.4 mmHg) after the observation period. As expected, cognitive level of patients with AD was significantly lower compared to CTRL (Table 3). AD patients did not exhibit further decreases in MMSE during the 12-month of standard institutionalized diet. N_MED_ taken by the patients with AD was significantly higher compared to CTRL (Table 3). Specifically, AD patients were treated with Quetiapine (32±7%), Citalopram (26±4%), Donepezil (6±2%), Memantine (2±1%), Ticlopidin (12±4%), Furosemide (15±3%). Additionally, the energy intake calories calculated from the daily eaten meals, and the adherence to the diet program (Fig 2), indicated a similar daily energy ingestion in AD patients and CTRL (Table 3). Interestingly, DEE, divided in PA and RMR, was similar in both groups (Table 3).

**Table 2:** Hematological and blood chemistry measurements at baseline (PRE) and after 12 months (POST) of controlled diet.

Measure	CTRL		AD	
	PRE (n. 86)	POST (n. 83)	PRE (n. 85)	POST (n. 80)
Glucose (mg∙dl^-1^)	88 ± 4	88 ± 4	87 ± 3	87 ± 3
RBC (10^6^∙μl^-1^)	3.90 ± 0.26	3.96 ± 0.19	3.96 ± 0.21	3.95 ± 0.20
Hb (g∙dl^-1^)	12.9 ± 0.88	13.3 ± 0.92	12.8± 0.84	13.1± 0.85
HDL (mg∙dl^-1^)	51.8 ± 1.4	52.3 ± 1.4	51.9 ± 1.2	51.8 ± 1.0
LDL (mg∙dl^-1^)	98.3 ± 2.7	97.9 ± 2.7	98.5 ± 3.0	97.9 ± 3.1
Creatinine (mg∙dl^-1^)	0.67 ± 0.09	0.66 ± 0.09	0.66 ± 0.09	0.65 ± 0.07
Azotemia (mg∙dl^-1^)	34.6 ± 5.44	34.3 ± 4.95	34.8 ± 5.53	36.9 ± 5.66
Na^+^ (mEq∙l^-1^)	142.6 ± 3.10	142.3 ± 3.30	142.3 ± 3.75	142.0 ± 3.11
K^+^ (mEq∙l^-1^)	3.80 ± 0.43	3.73 ± 0.44	3.74 ± 0.46	3.73 ± 0.42
Cl^-^ (mEq∙l^-1^)	101.8 ± 3.13	102.2 ± 3.03	102.1 ± 3.44	102.1 ± 3.06
AST (U∙l^-1^)	32.8 ± 12.7	29.8 ± 13.9	30.3 ± 12.9	30.5 ± 13.2
ALT (U∙l^-1^)	25.7 ± 9.8	25.2 ± 10.1	26.1 ± 10.8	23.6 ± 10.3
GTP (U∙l^-1^)	19.9 ± 7.0	19.9 ± 7.6	19.9 ± 7.6	19.1 ± 6.9
Bilirubin (mg∙dl^-1^)	0.67 ± 0.23	0.61 ± 0.24	0.61 ± 0.20	0.65 ± 0.22
Bilirubin dir (mg∙dl^-1^)	0.20 ± 0.07	0.21 ± 0.08	0.18 ± 0.07	0.20 ± 0.08
Bilirubin fra (mg∙dl^-1^)	0.47 ± 0.23	0.39 ± 0.27	0.43 ± 0.21	0.44 ± 0.21

**Table 3:** Inflammation, antioxidant carrier, nutritional state, cardiovascular, cognitive, functional state, number of medications, energy intake, daily energy expenditure, resting metabolic rate (RMR), and energy consumed by physical activity (PA) at baseline (PRE) and after 12 months (POST) of controlled diet.

Measure	CTRL		AD	
	PRE (n. 86)	POST (n. 83)	PRE (n. 85)	POST (n. 80)
WBC (n∙μl^-1^)	6102 ± 1151	5909 ± 1109	8670 ± 1129 †	8597 ± 1044 †
Neutrophil (% of total)	55.3 ± 5.8	54.3 ± 6.0	67.7 ± 4.5 †	66.3 ± 7.9 †
Eosinophil (% of total)	3.00 ± 1.63	3.20 ± 1.80	3.65 ± 1.65	3.51 ± 1.47
Basophil (% of total)	0.96 ± 0.83	0.97 ± 0.82	0.97± 0.84	0.94± 0.78
Lymphocytes (% of total)	28.3 ± 4.3	27.1 ± 5.0	26.9 ± 4.5	27.1± 4.5
Mon. mac. (% of total)	5.9 ± 2.6	5.9 ± 2.4	5.8 ± 2.3	6.5± 2.4
Albumin (g∙dl^-1^)	4.38 ± 0.30	4.38 ± 0.26	3.00 ± 0.53 †	2.97 ± 0.57 †
Retinol (μg∙dl^-1^)	45.8 ± 1.9	45.5 ± 2.0	45.1 ± 2.3	45.5 ± 1.8
Systolic BP (mmHg)	127 ± 9	126 ± 7	132 ± 9 †	131 ± 9 †
Diastolic BP (mmHg)	82 ± 3	82 ± 2	81 ± 4	80 ± 4
MMSE (0–30)	28 ± 2	28 ± 2	13 ± 2 †	12 ± 2 †
Barthel (0–100)	49 ± 8	50 ± 9	52 ± 9	51 ± 9
Balance test (0–16)	12 ± 2	12 ± 2	13 ± 2	13 ± 2
Gait test (0–12)	10 ± 1	10 ± 1	10 ± 1	10 ± 1
N_MED_	3 ± 1	3 ± 1	6 ± 1 †	6 ± 1 †
Energy intake (kcal⋅24h^-1^)	1804 ± 157	1810 ± 163	1804 ± 159	1812 ± 140
Energy expenditure (kcal⋅24h^-1^)	1629 ± 153	1614 ± 152	1640 ± 132	1610 ± 128
RMR (kcal⋅24h^-1^)	1059 ± 99	1049 ± 102	1066 ± 86	1046 ± 83
PA (kcal⋅24h^-1^)	570 ± 54	564 ± 53	574 ± 46	563 ± 45

† P<0.05 *vs* CTRL.

### Predictors of BM changes in patients with AD

Secondary outcomes assessed at baseline in patients with AD are represented with age- and sex-adjustment in Table 4. The 55 W with AD were significantly different in comparison to the 25 M with AD in terms of BM, number of red blood cells, systolic blood pressure, behavioral disturbance, salivary levels of cortisol, and daily energy intake. No difference in secondary outcomes, expect for the the duration of disease, has been detected between patients with AD adjusted for age (Table 4).

**Table 4:** Baseline characteristics of patients with AD adjusted by sex (women (W); men (M)) and age (less than 75 yrs; between 75 and 80 yrs; more than 80 yrs).

Measure	AD_sex_		AD_age_		
	W (n. 55)	M (n. 25)	<75 yrs (n. 29)	75–80 yrs (n. 32)	>80 (n. 16)
Age (yrs)	77 ± 5	76 ± 4	72 ± 2	77 ± 2	83 ± 2
Body mass (kg)	57.8 ± 5.5	69.9 ± 4.1 *	61.0 ± 8.1	62.8 ± 6.9	60.2 ± 7.7
Duration of disease (yrs)	8 ± 2	7 ± 2	6 ± 2	7 ± 2	10 ± 1 §
N_MED_ (n)	6 ± 1	6 ± 1	4 ± 1	4 ± 1	4 ± 1
N_comorbit_ (n)	2 ± 1	2 ± 1	2 ± 1	2 ± 1	2 ± 1
WBC (n∙μl^-1^)	8684 ± 1122	8733 ± 1145	8761 ± 1158	8815 ± 1173	8367 ± 942
ALB (g∙dl^-1^)	3.01 ± 0.55	3.03 ± 0.39	2.97 ± 0.47	3.04 ± 0.42	3.03 ± 0.68
Retinol (μg∙dl^-1^)	45.4 ± 2.3	44.9 ± 2.4	45.3 ± 2.2	44.8 ± 2.6	45.8 ± 1.8
Glucose (mg∙dl^-1^)	87 ± 3	87 ± 3	87 ± 3	87 ± 3	88 ± 4
RBC (10^6^∙μl^-1^)	3.92 ± 0.24	4.02 ± 0.13 *	3.99 ± 0.17	3.96 ± 0.25	3.86 ± 0.20
Hb (g∙dl^-1^)	12.9 ± 0.9	12.9 ± 0.9	12.7 ± 0.8	12.9 ± 0.8	13.0 ± 0.9
HDL (mg∙dl^-1^)	51.9 ± 1.2	52.1 ± 1.2	52.2 ± 1.3	51.7 ± 0.9	52.1 ± 1.3
LDL (mg∙dl^-1^)	98.0 ± 3.1	98.8 ± 2.9	97.8 ± 1.4	98.1 ± 3.5	99.2 ± 4.0
SBP (mmHg)	134 ± 10	128 ± 8 *	132 ± 11	132 ± 8	134 ± 10
DBP (mmHg)	81 ± 5	80 ± 3	81 ± 4	80 ± 5	81 ± 4
MMSE (0–30)	13.1 ± 1.7	13.6 ± 1.6	13.3 ± 1.6	13.3 ± 1.8	13.1 ± 1.7
Barthel (0–100)	53 ± 9	50 ± 10	53 ± 10	50 ± 9	53 ± 8
Behavioral disturbance (NPI)	63 ± 19	76 ± 17 *	69 ± 19	68 ± 22	63 ± 15
COR (nmol∙l^-1^)	9.5 ± 2.0	10.5 ± 1.6 *	9.9 ± 2.0	9.7 ± 2.1	9.7 ± 1.2
Balance test (0–16)	13 ± 2	12 ± 1	13 ± 2	13 ± 2	13 ± 2
Gait test (0–12)	10 ± 1	10 ± 1	10 ± 1	10 ± 1	10 ± 1
Energy intake (kcal⋅24h^-1^)	1775 ± 161	1859 ± 137 *	1799 ± 163	1765 ± 147	1875 ± 152
Energy expenditure (kcal⋅24h^-1^)	1637 ± 144	1640 ± 120	1644 ± 117	1629 ± 144	1656 ± 153
RMR (kcal⋅24h^-1^)	1065 ± 93	1066 ± 78	1078 ± 77	1058 ± 93	1038 ± 99
PA (kcal⋅24h^-1^)	573 ± 50	574 ± 42	579 ± 41	570 ± 50	581 ± 53

Number of medications taken (N_MED_); number of comorbidities (N_comorbit_); white blood cells (WBC); plasma level of albumin (ALB); red blood cells (RBC); hemoglobin (Hb); high density lipoprotein (LDL); low density lipoprotein (LDL); systolic blood pressure (SBP); diastolic blood pressure (DBP); salivary level of cortisol (COR); cognitive function (MMSE); severity of behavioral disorders (NPI); resting metabolic rate (RMR); energy consumed by physical activity (PA).

* P<0.05 *vs* W

§ P<0.05 *vs* <75yrs.

The univariate Pearson’s correlation model applied to the secondary outcomes assessed at baseline in the groups of W and M with AD are displayed in Tables 5 and 6 respectively. In the group of W with AD the baseline secondary outcomes significantly correlated with ∆BM were the N_MED_, ALB, NPI, COR, and BM (Table 5). Conversely, in the group of M with AD the baseline values significantly correlated with ∆BM were the N_MED_, ALB, NPI, MMSE, and COR (Table 6). The forward stepwise regression models utilized for the calculation of the interaction between the significantly correlated secondary outcomes assed at baseline and the ∆BM in W and M with AD are displayed in Tables 7 and 8. The forward stepwise regression utilized for the prediction of ∆BM in W with AD revealed that the combination of 3 factors N_MED_, ALB, and COR can predict this clinical phenomenon (Table 7). While at contrary, the best predictor of ∆BM in M with AD was the COR (Table 8).

**Table 5:** Correlations between secondary outcomes assessed at baseline and changes in body mass (∆BM) in 55 W with AD. Duration of disease (Dur); number of medications taken (N_MED_); number of comorbidities (N_com_); systolic blood pressure (SBP); diastolic blood pressure (DBP); glucose (GLU); red blood cells (RBC); hemoglobin (Hb); white blood cells (WBC); high density lipoprotein (LDL); low density lipoprotein (LDL); plasma level of albumin (ALB); plasma level of retinol (Retinol); activity of daily living (Barthel); severity of behavioral disorders (NPI); salivary level of cortisol (COR); cognitive function (MMSE); body mass (BM); daily energy intake (DEI); daily energy expenditure (DEE); resting metabolic rate (RMR); energy consumed by physical activity (PA).

	Age	Dur	N_MED_	N_COM_	SBP	DBP	GLU	RBC	Hb	wbc	HDL	LDL	ALB	Retinol	Barthel	MMSE	NPI	COR	BM	DEI	DEE	RMR	PA	∆BM
**∆BM**	-0.168	-0.221	-0.671*	-0.045	0.015	-0.03	-0.067	0.101	-0.135	0.168	0.198	0.013	0.591*	-0.099	-0.178	0.062	0.366*	0.382*	0.438*	0.055	-0.101	-0.101	-0.101	1.
**PA**	-0.054	-0.037	0.113	0.263	-0.09	-0.213	0.154	0.086	0.115	-0.274*	0.019	0.061	0.066	-0.139	0.195	-0.012	0.104	0.09	0.057	0.604*	1.	1.	1.	
**RMR**	-0.054	-0.037	0.113	0.263	-0.09	-0.213	0.154	0.086	0.115	-0.274*	0.019	0.061	0.066	-0.139	0.195	-0.012	0.104	0.09	0.057	0.604*	1.	1.		
**DEE**	-0.054	-0.037	0.113	0.263	-0.09	-0.213	0.154	0.086	0.115	-0.274*	0.019	0.061	0.066	-0.139	0.195	-0.012	0.104	0.09	0.057	0.604*	1.			
**DEI**	0.232	0.115	0.15	0.154	-0.157	-0.107	0.118	0.087	0.133	-0.157	0.015	0.261	0.031	0.081	0.093	-0.135	0.015	0.042	0.282*	1.				
**BM**	0.172	0.26	0.406*	-0.041	0.175	-0.209	0.13	-0.109	0.034	-0.172	0.017	0.062	0.279*	-0.139	-0.023	-0.008	0.014	0.021	1.					
**COR**	0.158	0.1	0.082	-0.152	-0.057	0.32*	-0.267*	0.007	0.241	-0.037	0.174	0.02	0.244	0.207	0.226	0.023	0.785*	1.						
**NPI**	0.037	0.106	0.156	-0.038	-0.08	0.156	-0.139	0.197	0.079	-0.116	0.012	0.134	0.182	0.043	0.173	0.093	1.							
**MMSE**	-0.145	-0.255	-0.114	0.141	-0.187	-0.243	-0.164	-0.095	-0.14	0.152	0.156	0.018	0.059	-0.165	-0.025	1.								
**Barthel**	0.078	0.103	-0.152	-0.269*	-0.043	0.116	-0.03	0.03	0.091	-0.078	0.15	0.263	0.084	0.199	1.									
**Retinol**	0.094	0.156	0.033	-0.091	0.39*	0.299*	0.094	-0.175	0.114	0.147	0.073	0.008	0.093	1.										
**ALB**	-0.179	-0.081	-0.392*	0.014	0.121	-0.042	0.008	0.169	-0.094	0.2	0.034	0.063	1.											
**LDL**	0.049	0.057	-0.038	-0.297*	-0.048	-0.01	-0.052	-0.178	0.104	0.033	0.138	1.												
**HDL**	-0.025	0.226	-0.048	-0.029	0.16	-0.182	-0.044	-0.071	-0.087	-0.146	1.													
**wbc**	-0.237	-0.332*	-0.132	-0.182	-0.113	-0.023	0.002	0.161	-0.151	1.														
**Hb**	0.187	0.033	0.068	0.081	0.077	0.045	-0.034	-0.027	1.															
**RBC**	-0.253	-0.279*	0.098	0.074	-0.191	-0.17	-0.177	1.																
**GLU**	0.092	0.143	0.187	-0.062	0.254	0.105	1.																	
**DBP**	-0.024	0.013	-0.154	-0.223	0.203	1.																		
**SBP**	0.026	0.059	0.036	-0.169	1.																			
**N**_**COM**_	0.05	-0.023	0.01	1.																				
**N**_**MED**_	0.166	0.143	1.																					
**Dur**	0.741*	1.																						
**Age**	1.																							

Correlations are represented by r values

* P<0.05.

**Table 6:** Correlations between secondary outcomes assessed at baseline and changes in body mass (∆BM) in 25 M with AD. Duration of disease (Dur); number of medications taken (N_MED_); number of comorbidities (N_com_); systolic blood pressure (SBP); diastolic blood pressure (DBP); glucose (GLU); red blood cells (RBC); hemoglobin (Hb); white blood cells (WBC); high density lipoprotein (LDL); low density lipoprotein (LDL); plasma level of albumin (ALB); plasma level of retinol (Retinol); activity of daily living (Barthel); severity of behavioral disorders (NPI); salivary level of cortisol (COR); cognitive function (MMSE); body mass (BM); daily energy intake (DEI); daily energy expenditure (DEE); resting metabolic rate (RMR); energy consumed by physical activity (PA).

	Age	Dur	N_MED_	N_COM_	SBP	DBP	GLU	RBC	Hb	WBC	HDL	LDL	ALB	Retinol	Barthel	MMSE	NPI	COR	BM	DEI	DEE	RMR	PA	∆BM
**∆BM**	-0.009	-0.181	-0.593*	0.366	0.079	-0.258	0.077	0.036	0.081	-0.323	-0.135	0.151	0.466*	-0.314	-0.209	0.450*	-0.709*	-0.782*	-0.139	-0.235	0.009	0.009	0.009	1.
**PA**	0.069	-0.106	0.104	-0.196	-0.083	0.188	-0.149	-0.168	-0.143	-0.393	-0.287	0.430*	-0.038	-0.187	0.093	0.307	0.034	0.005	0.138	0.309	1.	1.	1.	
**RMR**	0.069	-0.106	0.104	-0.196	-0.083	0.188	-0.149	-0.168	-0.143	-0.393	-0.287	0.430*	-0.038	-0.187	0.093	0.307	0.034	0.005	0.138	0.309	1.	1.		
**DEE**	0.069	-0.106	0.104	-0.196	-0.083	0.188	-0.149	-0.168	-0.143	-0.393	-0.287	0.430*	-0.038	-0.187	0.093	0.307	0.034	0.005	0.138	0.30	1.			
**DEI**	0.024	0.103	-0.027	-0.073	-0.035	0.001	-0.073	-0.263	0.243	0.001	-0.186	0.132	-0.298	-0.078	0.143	0.141	0.176	0.087	-0.042	1.				
**BM**	-0.200	-0.148	0.265	-0.125	-0.221	-0.031	-0.163	0.390	-0.109	0.092	-0.197	-0.279	-0.110	0.288	0.084	0.162	0.106	-0.161	1.					
**COR**	-0.085	0.121	0.546*	-0.257	0.101	0.129	-0.052	-0.180	-0.300	0.414*	0.223	-0.097	-0.345	0.156	0.012	-0.389	0.746*	1.						
**NPI**	-0.285	-0.006	0.796*	-0.340	-0.078	-0.091	0.003	-0.088	-0.146	0.348	0.192	-0.120	-0.210	0.147	0.171	-0.125	1.							
**MMSE**	0.140	-0.109	-0.241	-0.095	-0.286	-0.310	0.019	0.065	-0.139	-0.156	-0.042	0.227	0.410*	-0.375	-0.335	1.								
**Barthel**	-0.108	0.084	0.195	-0.178	0.251	0.340	-0.067	-0.122	0.152	0.216	-0.066	0.011	-0.073	-0.092	1.									
**Retinol**	-0.013	-0.099	0.120	0.014	0.415*	0.329	0.139	-0.061	0.201	0.070	0.170	-0.211	-0.351	1.										
**ALB**	0.306	0.110	-0.102	0.242	0.019	-0.326	0.384	0.022	-0.067	-0.202	0.038	0.131	1.											
**LDL**	0.454*	0.255	-0.135	-0.267	0.177	0.299	-0.029	-0.101	0.062	-0.177	0.118	1.												
**HDL**	0.131	0.639*	0.018	-0.027	0.289	-0.173	0.360	0.173	0.092	0.252	1.													
**WBC**	0.018	0.160	0.113	-0.442*	-0.009	-0.034	-0.08	-0.113	0.030	1.														
**Hb**	0.266	0.344	-0.328	-0.105	0.158	0.151	0.206	-0.204	1.															
**RBC**	-0.293	0.039	0.110	0.258	-0.102	-0.030	-0.244	1.																
**GLU**	0.213	0.312	0.137	0.123	0.294	-0.238	1.																	
**DBP**	0.301	-0.06	-0.148	-0.175	0.431*	1.																		
**SBP**	-0.005	0.097	-0.124	0.235	1.																			
**N**_**COM**_	-0.054	0.042	-0.104	1.																				
**N**_**MED**_	-0.322	-0.091	1.																					
**Dur**	0.546*	1.																						
**Age**	1.																							

Correlations are represented by r values

* P<0.05.

**Table 7:** Forward stepwise regression of secondary outcomes assed at baseline which predict ∆BM in W with AD. number of medications taken (N_MED_); plasma level of albumin (ALB); salivary level of cortisol (COR).

*R*	*R-square*	*Adjusted R-square*	*S*	*F*	*p-level*		
0.7991	0.63855	0.61687	1.46772	29.44453	0.0000		
*VAR*	*Coefficient*	*Standard Error*	*Beta*	*t*	*p-level > t*	*VIF*	*TOL*
N_MED_	-1.10148	0.19398	-0.52329	-5.67832	0.00000	1.17484	0.85118
ALB	1.38111	0.40977	0.31952	3.37049	0.00145	1.24319	0.80438
COR	-0.30862	0.10327	-0.26234	-2.98855	0.00434	1.06596	0.93812
Intercept	0.20344						

**Table 8:** Forward stepwise regression of secondary outcomes assed at baseline which predict ∆BM in M with AD. salivary level of cortisol (COR).

Stepwise regression (M)							
R	R-square	Adjusted R-square	S	F	p-level		
0.78248	0.61227	0.59541	1.53055	36.31949	0.0000		
VAR	Coefficient	Standard Error	Beta	t	p-level > t	VIF	TOL
COR	-1.20991	0.20076	-0.78248	-6.02657	0.000000	1.	1.
Intercept	10.76826						

## Discussion

**Fig 5:**
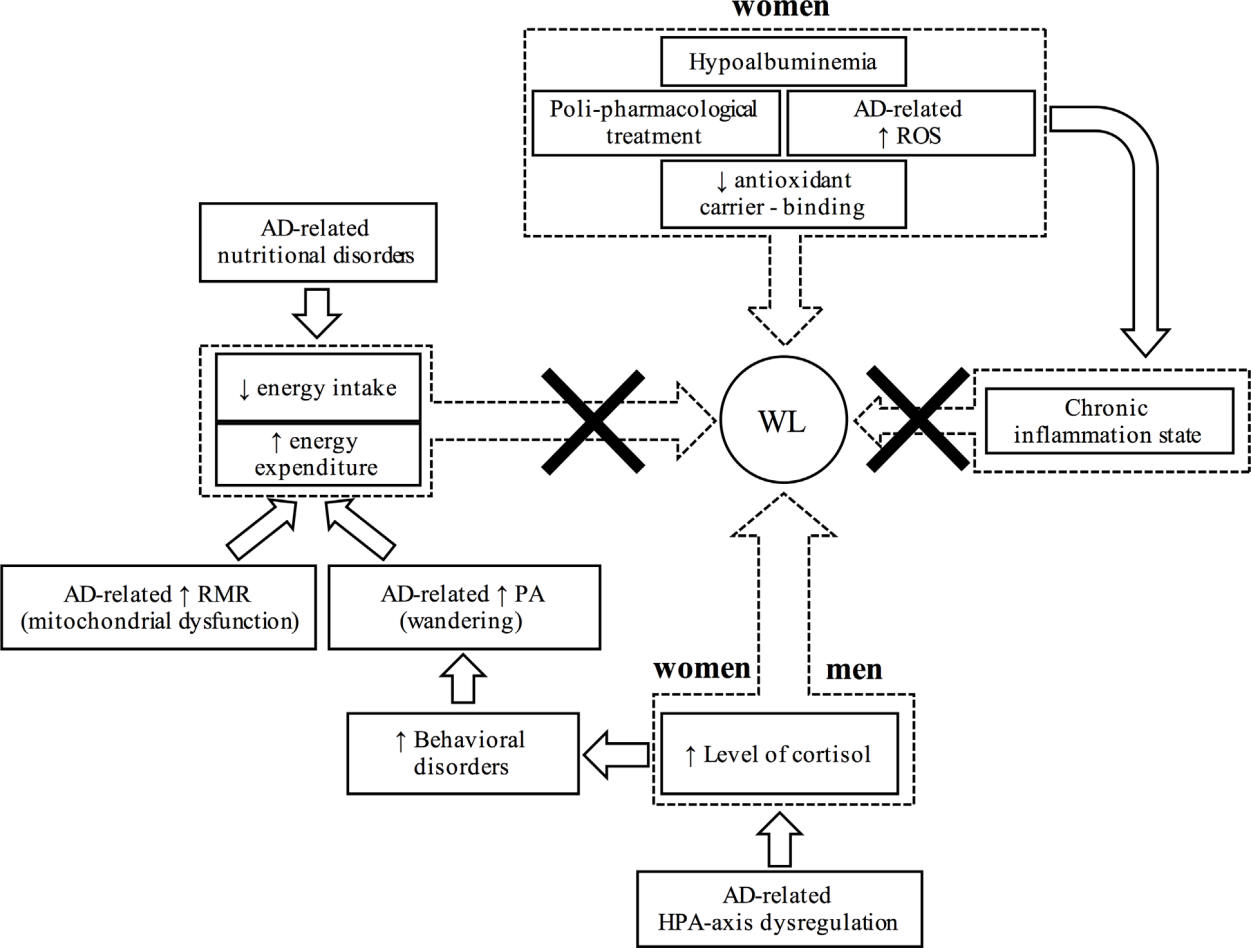
Causal mechanisms linking Alzheimer’s disease (AD) and involuntary weight loss (WL). Resting metabolic rate (RMR); daily physical activity (PA); hypothalamic–pituitary–adrenal axis (HPA-axis); reactive oxygen species (ROS).

The main finding of the present cohort study was that involuntary WL, determined by BM reduction in patients with AD was not influenced by patients’ energy intake. Additionally, our data of DEE suggest that the changes in BM of patients with AD were not affected by their metabolism rate. Conversely, the combination of others factors, i.e., hypoalbuminemia, number of medications, and hypercortisolism, contributed to the involuntary WL in the present group of W with AD. Conversely, data of M with AD followed in this cohort study indicate that WL was primarily correlated with the baseline values of salivary cortisol. Indeed, involuntary WL in patients with AD is a multifaceted clinical phenomenon, and in the current cohort study we were able to establish, at list in part, some of the factors associated with involuntary WL. The overall plausible causal mechanisms linking AD and WL, including the potential pathways and the biomarkers utilized in this study, are displayed in the schematic Fig 5.

### Energy intake and involuntary WL

AD is typically associated with nutritional disorders [6]. It has been demonstrated that the WL usually exhibited by old individuals with dementia can be attenuated by increasing their energy intake [3]. However, Singh et al. [21] reported that WL in patients with AD was not accompanied by any apparent deficit in food intake or malabsorption. Accordingly, data from the current cohort study indicate that even in a condition of controlled standard institutionalized diet, the present groups of W and M with AD suffered of a remarkable WL. Noticeably, at baseline and after the 12-month of standard institutionalized diet, the blood level of retinol was similar in the two groups, implicating the adequacy of the energy intake adopted in this cohort study. Thus, unilateral approaches based upon controlled standard institutionalized diets seem not to be adequate to prevent and control this clinical phenomenon (Fig 5).

### Metabolic rate and involuntary WL

In previous investigations, the potential association between metabolism, AD and WL has been investigated, since a disproportion between energy intake and energy expenditure could produce WL [6]. In AD individuals a high energy expenditure could be induced by an elevated RMR, and by the increased levels of PA due to behavioral disorders, such as wandering. Interestingly, Knight et al. [4] examined the longitudinal variations of BM, food intake, and metabolic rate in a triple-transgenic mouse model of AD, and revealed a raised RMR. However, when moving into investigations involving humans, such results are controversial [5, 22]. Data from the current study indicate a RMR similar between individuals with AD and controls, and no correlation between RMR and involuntary WL in both W and M with AD. Thus, in agreement with the literature on humans [5, 23], it seems that the BM changes cannot be ascribed to an elevated RMR. Moreover, wandering, a typical clinical sign of aberrant motor behavior in patients with AD has been hypothesized to increase levels of PA, thus possibly affect BM changes [6]. Conversely, Secher et al. [24] revealed that AD patients with elevated behavioral disturbances even gained body weight. In addition, Poehlman et al., [23] observed reduced levels of PA in patients with AD compared to healthy elderly controls. The results of the present cohort study indicate that the energy expenditure related to PA was not different between individuals with AD that suffered of behavioral disturbances, and age-matched controls without dementia (Table 3). Moreover, the poor correlation between PA and ∆BM in W and M with AD (Tables 5 and 6) suggest that the etiology of WL observed in patients with AD is likely influenced by other factors (Fig 5).

### Hypoalbuminemia and involuntary WL

The well-recognized body composition changes in patients with AD are similar, yet more severe, to the standard age-related sarcopenia. All the most credited theories of aging agree that age-related accumulation of reactive oxygen species is a main contributor of cellular senescence, and this factor clearly contribute to the skeletal muscle atrophy [25, 26]. In this context, the literature highlights a close correlation between the levels of reactive oxygen species and AD [11]. Moreover, Kim et al. [12] indicated that the major plasma carrier of antioxidants, albumin, was significantly reduced in patients with AD. In agreement, results of the current cohort study suggest that in W with AD a reduction of plasma levels of albumin, in combination with the chronic poli-pharmacological treatment adopted (that additionally reduces antioxidant binding capacity of albumin), significantly affected BM (Fig 5). Therefore, particular attention should be taken in the evaluation of the plasma levels of albumin in W with AD, because this parameter may predict a possible reduction of BM.

### Chronic inflammation state and involuntary WL

It has been reported that peripheral leukocytes are altered in patients with AD [27]. Remarkably, the elevation in oxidative stress markers is also associated with an increased number of neutrophils [13]. Therefore, it may be reasonably expected a role of the chronic inflammation in the BM changes in patients with AD. However, the results from the current cohort study suggest that the chronic inflammation state, demonstrated by the elevated levels of neutrophils, was not correlated to the 12-month changes in BM for both W and M with AD. Therefore, the relevance of the chronic inflammation in the development of body composition changes in patients with AD does not seem to be crucial (Fig 5).

### Poli-pharmacological treatment and involuntary WL

Patients with AD are chronically treated with several medications, and the potential effect of these drugs on WL has been already investigated. For instance, in previous studies [7, 24] the effect of cholinesterase inhibitors on WL in patients with AD has been debated, but with controversy. In addition, pharmacological interactions among medications may potentially exacerbate the side effects or may produce malabsorption of macro and micro nutriments thus influencing body composition. Interestingly, the number of drugs daily taken by both W and M with AD in this study was correlated to WL. This circumstance, in combination with the simultaneous diminished albumin plasma levels, likely reduced the bulk delivery of antioxidants (Fig 5). Therefore, attention should be taken for the prescription of additional medications, with particular focus on the total number of drugs daily taken by patients.

### Hypercortisolism and involuntary WL

HPA-axis activity is altered in patients with AD [9, 28]. The effects of such dysregulation are wide-ranging, but the most evident consequence is an increase in cortisol levels. The hypercortisolism in patients with AD is correlated with the exacerbation of behavioral disorders, such as the sundowining syndrome [9, 29]. Elevated levels of cortisol, one of the most catabolic hormones, lead also to a remarkable sarcopenia. Interestingly, data from the current cohort study confirm that in both W and M with AD the cortisol levels were significantly higher (Fig 4). Noticeably, these elevated values of cortisol were significantly correlated (r = 0.785 and r = 0.746) with the severity of behavioral disorders estimated with the NPI test (Tables 5 and 6) for W and M with AD respectively. Most importantly, the BM changes in 12 months were significantly correlated with the elevated cortisol levels (Tables 5 and 6; Fig 5). Therefore, the cortisol levels, which are not routinely assessed in patients with AD, would help to predict patients at risk of WL.

### Predictors of involuntary WL in W and M with AD

Our data has revealed that sex was a potential covariant for ∆BM, in W and M with AD. Therefore, to establish potential single correlation between each baseline parameters and ∆BM in W and M with AD univariate Pearson’s correlation models were performed. Secondary outcomes assessed at baseline that were significantly correlated with ∆BM have been considered as potential predictors of involuntary WL for W and M with AD (Tables 5 and 6). Moreover, the result of the forward stepwise regression model suggests that ∆BM in W with AD (Table 7) can be predicted by the combination of three factors: N_MED_, ALB, and COR. For instance, a W with AD that is treated with 4 different medications, have ALB values of 3.03 g∙dl^-1^, and salivary COR levels of 9.5 nmol∙l^-1^ will probably lose ∼3 kg of BM during one year of her institutionalized life. Conversely, WL in M with AD appears to be mainly related to the COR levels (Table 8). In this case, a M with AD with a salivary COR levels of 10.6 nmol∙l^-1^ will probably lose ∼2 kg of BM during one year of his institutionalized life.

## Conclusion

Patients with AD that suffered of involuntary WL are at high risk of frailty and mortality. This phenomenon is multifactorial and the etiology could be related to the inadequacy of energy intake. However, the results of the current cohort study highlighted the critical role of the hypoalbuminemia, the number of medications taken, and the hypercortisolism, in BM changes in W with AD. Conversely, involuntary WL in M with AD appears mainly associated with hypercortisolism. It is important to note, that data from the current study, especially taking into account energy expenditure values, are based upon institutionalized individuals and such results may not be applied to community living elderly.

Nevertheless, the assessment of these values in a clinical milieu, and their correct utilization into the equations for W and M with AD extrapolated from the current investigation, may be helpful in predicting this challenging clinical phenomenon.

## Supporting Information

S1 File STROBE Checklist. (DOCX)
